# Microsatellite markers for *Saussurea polylepis* (Asteraceae), a vulnerable continental island species endemic to Korea

**DOI:** 10.1002/aps3.11270

**Published:** 2019-06-14

**Authors:** Seon A Yun, Seung‐Chul Kim

**Affiliations:** ^1^ Department of Biological Sciences Sungkyunkwan University Seobu‐ro 2066 Suwon 16419 Korea

**Keywords:** Asteraceae, conservation, continental islands, microsatellite markers, *Saussurea polylepis*

## Abstract

**Premise:**

Nuclear microsatellite markers were developed for *Saussurea polylepis* (Asteraceae), a vulnerable species with very limited distribution in a few southwestern continental islands of the Korean peninsula, in order to facilitate future population genetic studies.

**Methods and Results:**

Based on the Illumina sequence data, a total of 21 microsatellite primer pairs were designed and tested for their suitability. Nineteen of these primers, with two to 11 alleles per locus, were polymorphic in three natural populations of *S. polylepis*. The levels of expected and observed heterozygosity ranged from 0.000 to 0.842 and 0.000 to 0.933, respectively. Sixteen of these simple sequence repeat (SSR) markers were successfully cross‐amplified in five congeneric species, namely *S. gracilis*,* S. grandifolia*, and *S. tanakae* for all 21 loci, and *S. maximowiczii* and *S. pulchella* for 18 loci.

**Conclusions:**

The SSR markers developed here will be useful for future population genetic studies on *S. polylepis* and related species.

*Saussurea* DC. (ca. 400 species) is one of the largest genera in the tribe Cardueae (Asteraceae) and is also one of the most speciose groups in the flora of Korea (Lipschitz, 1979; Im, 2007). Of the approximately 40 species of *Saussurea* in Korea, nearly 40% (15 species) are endemic, mostly occurring in small‐sized populations in isolated regions. Of these endemic species, *S. polylepis* Nakai, a perennial herb, is highly restricted to small continental islands (less than 10 locations) in the southwestern corner of the Korean peninsula. Its population size is extremely small, normally less than 30 individuals per population, and it is currently categorized as a vulnerable species (VU B2ab (iii, iv)) in the Korean Red List (National Institute of Biological Resources, 2014). Although the overall phylogenetic framework among the species of *Saussurea* in Korea is lacking, *S. polylepis* can be morphologically distinguished from its congeneric species by its glossy and reniform middle cauline leaves, hairs on adaxial and abaxial leaf surfaces, petioles without wings, and irregularly dentate leaf margins (Im, 2007).

*Saussurea* includes numerous ecologically and ethnomedicinally important species. For example, some species, such as *S. lappa* (Decne.) Sch. Bip., *S. gnaphalodes* (Royle ex DC.) Sch. Bip., *S. costus* (Falc.) Lipsch., and *S. ceratocarpa* Decne., contain many important chemical compounds that have become important drugs in the international market (Pandey et al., 2007; Zeng et al., 2012). In addition, some species occur at higher elevations than any vascular plant (e.g., *S. gnaphalodes* in the vicinity of Mount Everest at 6400 m; Yoshida, 2002; Yang et al., 2008). Because of their distribution in the subalpine/alpine regions and overexploitation for different medicinal and commercial purposes, many of these species are sensitive to climatic change and are subject to extinction. Several endemic species of *Saussurea* in the backbone mountain range (i.e., Baekdudaegan) in the Korean peninsula are not an exception to this situation (e.g., *S. grandicapitula* W. Lee & Im, *S. chabyoungsanica* Im, and *S. calcicola* Nakai). Despite the ecological and economic importance of the genus *Saussurea*, very few studies on population genetics and molecular marker development have been conducted (Jeong et al., 2012; Zeng et al., 2012). In this study, we selected one very rare species, *S. polylepis*, occurring in several continental islands in Korea, and developed polymorphic microsatellite markers for future population genetic studies. In addition, we tested whether some of these markers could be cross‐amplified to five closely related species of *Saussurea*, namely *S. gracilis* Maxim., *S. grandifolia* Maxim., *S. maximowiczii* Herder, *S. pulchella* (Fisch.) Fisch. ex Colla, and *S. tanakae* Franch. & Sav. ex Maxim., which are commonly found in mountain regions of Korea.

## METHODS AND RESULTS

Total genomic DNA was extracted from a fresh leaf sample collected in Hong‐do, Korea (Appendix 1), and deposited in the National Center for Biotechnology Information (NCBI) BioSample database (BioSample accession number SAMN10362162) using a DNeasy Plant Mini Kit (QIAGEN, Carlsbad, California, USA), following the manufacturer instructions. An Illumina paired‐end genomic library was constructed using a TruSeq DNA LT Sample Prep Kit (Illumina, San Diego, California, USA), and the library sequencing was conducted with the Illumina MiSeq platform at the Macrogen Company (Seoul, Korea). A total of 15,210,742 paired‐end reads (4.5 Gbp, NCBI Sequence Read Archive BioProject ID PRJNA503488) were obtained. The raw reads were trimmed to remove low‐quality bases using Sickle (Joshi and Fass, 2011) and adapters using Scythe (v0.994 BETA) (Buffalo, 2014). The paired‐end reads were then assembled using SPAdes version 2.4 (Bankevich et al., 2012), and a total of 814,626 contigs were obtained. MIcroSAtellite identification software (MISA) with default settings (Thiel et al., 2003) was used to detect simple sequence repeat (SSR) motifs with a repeat unit ranging from two to six nucleotides. The minimum number of nucleotide repeats was set to six for dinucleotide repeats and five for trinucleotide, tetranucleotide, pentanucleotide, and hexanucleotide repeats, and a total of 40,773 regions were found. Primer3 version 2.3.6 software (Koressaar and Remm, 2007; Untergasser et al., 2012) was used to design SSR primers with the following settings: optimal conditions of length of 20 bp (18–27 bp), annealing temperature of 60°C (57–63°C), and product size range of 100–300 bp. Of the 40,773 regions, 35 candidate primer pairs were randomly selected and tested for amplification efficiency using eight individuals of *S. polylepis* sampled from three island populations of Hueksan‐do, Hong‐do, and Ui‐do (Appendix 1). The PCR amplifications were performed in a total volume of 20 μL containing 1 μL of genomic template DNA, 0.5 μL of 10 mM forward primer with fluorescent dye (Table 1), 0.5 μL of 10 mM reverse primer, 2.5 μL (10×, with 2.5 mM MgCl_2_) of PCR buffer, 0.5 μL (each 10 mM) of dNTPs, and 0.1 μL (1.25 U/μL) of *Taq* DNA polymerase (Inclone Biotech, Gyeonggi‐do, Korea). The PCR conditions were: initial denaturation at 95°C for 1 min; followed by 35 cycles at 95°C for 20 s, annealing at 58–60°C for 40 s, and extension at 72°C for 60 s; and a final extension at 72°C for 5 min. The PCR amplicons were then separated with a GeneScan 400 LIZ Size Standard (Applied Biosystems, Foster City, California, USA) at the Macrogen Company utilizing an ABI 3730XL DNA Sequencer (Applied Biosystems). The resulting electropherograms were scored using GeneMapper v5.0 (Applied Biosystems). Of the 35 tested primer pairs, 21 produced repeatable amplicons and 19 showed polymorphisms across all eight individuals. These 19 primer pairs were subsequently scored using 48 individuals of *S. polylepis* sampled from the same three island populations and five individuals from each of the other species, namely *S. gracilis*,* S. grandifolia*,* S. maximowiczii*,* S. pulchella*, and *S. tanakae* (Appendix 1), for congeneric cross‐transferability of the markers. The PCR thermocycling conditions and genotyping method were the same as described above.

**Table 1:** Characteristics of 21 microsatellite markers developed for *Saussurea polylepis*

Locus	Primer sequences (5′–3′)	Repeat motif	Allele size range (bp)	*T*_a_ (°C)	Fluorescent dye	GenBank accession no.
SP01	F: TCACAAGCCCATTCAGCCAT	(CT)_10_	237–249	59	FAM	MK285611
	R: TACAGGCGACCCAAAGTGTC					
SP02	F: CAAGGCCGGAGCTTTTCGTA	(TC)_11_	269–273	59*, 60	HEX	MK285612
	R: TCCATTCGCACCGCTTCTTA					
SP03	F: CTGCCGGAGTCTGCTTCTAG	(AAT)_10_	234–297	59	FAM	MK285613
	R: TTTGGTCTCACACCCCTTGG					
SP04	F: CCGAACCCGTACTACAACCC	(AGT)_5_	225–234	60	HEX	MK285614
	R: TGCCAAGAGATCAACTAGGACG					
SP06	F: CTCAAGGAGTGGAAACCCCC	(AC)_10_	233–247	59	FAM	MK285615
	R: AAAAGGTCGGGGTCTCAACC					
SP07	F: CTCTGCAAGGATCGCTCCAA	(GA)_11_	233–287	60	FAM	MK285616
	R: CAATCGAGGCCCTAACCGAA					
SP10	F: GTTTTCCGGCCTCAACCAAC	(GGA)_7_	222–231	60	FAM	MK285610
	R: AACAATCCGGTAGCTGCCTC					
SP11^a^	F: CACGACAGGTTGGACGAAGA	(TCA)_9_	191, 200	59*, 60	HEX	MK285617
	R: CTGAGGGGAGCAACTCGAAG					
SP12	F: AGCGATTCAGATGTTGCCTCT	(TTG)_9_	268–295	58*, 59	FAM	MK285618
	R: ACAACATGTGGTTTCTCATGGT					
SP13	F: GCATACCGTCCCGATGAAGT	(TCTA)_8_	222–246	59	FAM	MK285619
	R: CCATCCCATGAATTGCGCAT					
SP19^b^	F: CTGCTCAGGATGTTCAAGAAGA	(GAT)_6_	249	58*, 59	HEX	MK285620
	R: CCGACATGGTAGCTTCCGAA					
SP20	F: GCTTCTCTACTCTTCGCCACA	(TAG)_7_	223–250	59	FAM	MK285621
	R: GTTTTCAGGGCCAGCCTTTG					
SP21	F: TGCCTCAACCTGATCGATCG	(TCT)_8_	253–256	59	FAM	MK285622
	R: TGACCAACCTTGTGCCAGAA					
SP22	F: TCAACAACCCCGACGAACAT	(TGTT)_6_	273–285	60	HEX	MK285623
	R: CCTCTCATTTTGTGCACCATGG					
SP23	F: ACCAGATTGTAGCACCCTCA	(ATAC)_7_	272–348	58*, 59	HEX	MK285624
	R: CGCACATTTGGAGATAGCCG					
SP25	F: GAACAACAAGGTTTTCCGGCA	(ATAC)_7_	253–265	59	FAM	MK285625
	R: GCCTCAGTTTCTCATGCCTA					
SP26	F: GCCCTAGCCTTTCTTGATGGA	(GC)_6_	237–239	59	FAM	MK285626
	R: CGACTGCGCTACATAGGGTT					
SP29	F: TGTCGCGCAAGTCTTCATCT	(GTT)_7_	267–276	59*, 60	HEX	MK285627
	R: CAATAGCCGACTGACTCGCT					
SP31	F: ACTGAATCGCTGCTTCTGCT	(ATAG)_8_	225–253	59*, 60	FAM	MK285628
	R: TGTCCATTCATGCTCTTTGTCA					
SP34	F: TGCTAGCAACAACGACTTGT	(TAAA)_6_	213–281	59*, 60	HEX	MK285629
	R: GATGTACCGCACTGCATTGC					
SP35	F: TTGGTGGAAGGCATGATGGA	(CATA)_10_	202–242	59	FAM	MK285630
	R: TCCATGGATGTTTTTGCATTGGT					

Note

*T*_a_ = annealing temperature.

a Fixed heterozygous locus.

b Monomorphic locus.

*Although most of the samples were amplified based on a higher *T*_a_ setting, some samples failed to amplify and thus lowered annealing temperatures were used for PCR.

Population genetic diversity parameters, including number of alleles, expected heterozygosity, and observed heterozygosity, were calculated using GenAlEx v6.502 (Peakall and Smouse, 2006). Deviations from Hardy–Weinberg equilibrium were calculated for each locus with GENEPOP 4.2 (Rousset, 2008). Of the 21 SSRs developed in this study, 19 were polymorphic. The expected and observed heterozygosity values ranged from 0.000 to 0.842 and from 0.000 to 0.933, respectively (Table 2). Of the 19 polymorphic loci, five in the Hong‐do population, five in the Heuksan‐do population, and three in the Ui‐do population deviated from Hardy–Weinberg equilibrium (Table 2).

**Table 2:** Genetic properties of 21 newly developed microsatellite markers in three populations of *Saussurea polylepis*.^a^

Locus	Hong‐do (*N* = 13)^b^			Heuksan‐do (*N* = 20)			Ui‐do (*N* = 15)		
	*A*	*H*_o_	*H*_e_	*A*	*H*_o_	*H*_e_	*A*	*H*_o_	*H*_e_
SP01	2	0.385	0.311	4	0.650	0.561	4	0.533	0.522
SP02	3	0.231	0.210	3	0.350	0.436	4	0.533	0.578
SP03	10	0.385	0.831*	10	0.350	0.811*	9	0.467	0.82*
SP04	3	0.231	0.210	3	0.250	0.224	5	0.467	0.482
SP06	3	0.615	0.494	7	0.750	0.789	7	0.733	0.678
SP07	10	0.769	0.820	11	0.850	0.785	10	0.933	0.842
SP10	3	0.615	0.618	3	0.700	0.660	3	0.533	0.604
SP11	2	1.000	0.500*	2	1.000	0.500*	2	1.000	0.500*
SP12	4	0.615	0.464	6	0.550	0.673	4	0.400	0.389
SP13	7	0.308	0.781*	6	0.500	0.735*	5	0.143	0.763*
SP19	1	0.000	0.000	1	0.000	0.000	1	0.000	0.000
SP20	1	0.000	0.000	3	0.100	0.096	1	0.000	0.000
SP21	1	0.000	0.000	2	0.050	0.049	3	0.133	0.127
SP22	2	0.231	0.500	3	0.400	0.434	2	0.133	0.124
SP23	7	0.385	0.808*	10	0.500	0.779*	10	0.733	0.838
SP25	3	0.462	0.530	4	0.400	0.468	4	0.667	0.633
SP26	2	0.077	0.311	2	0.050	0.349*	2	0.067	0.278
SP29	3	0.462	0.544	4	0.450	0.623	4	0.800	0.611
SP31	5	0.308	0.278	4	0.150	0.143	5	0.600	0.482
SP34	2	0.385	0.311	3	0.150	0.224	4	0.200	0.382
SP35	4	0.308	0.568*	8	0.700	0.834	4	0.267	0.429

Note

*A* = number of alleles; *H*_e_ = expected heterozygosity; *H*_o_ = observed heterozygosity; *N* = number of individuals.

a Locality and voucher information are provided in Appendix 1.

b Due to very small population size, 13 individuals were sampled.

*Significant deviation from Hardy–Weinberg equilibrium (*P* < 0.01).

**Table 3:** Cross‐amplification of 21 microsatellite loci developed for *Saussurea polylepis* in five related congeneric species.^a^

Locus	*S. grandifolia* (*N* = 5)		*S. maximowiczii* (*N* = 5)		*S. pulchella* (*N* = 5)		*S. tanakae* (*N* = 5)		*S. gracilis* (*N* = 5)	
	*A*	Allele size range (bp)	*A*	Allele size range (bp)	*A*	Allele size range (bp)	*A*	Allele size range (bp)	*A*	Allele size range (bp)
SP01	7	231–245	3	281–285	1	197	2	237–239	3	237–241
SP02	5	263–285	3	281–309	4	283–293	3	271–275	4	261–279
SP03	5	258–315	1	258	1	258	5	258–297	5	258–297
SP04	5	201–252	0	—	0	—	1	225	4	222–237
SP06	7	231–255	4	239–251	5	235–251	6	235–249	6	217–251
SP07	6	225–267	3	223–235	0	—	7	223–255	8	227–265
SP10	3	222–231	3	222–231	2	222–225	3	216–225	4	222–231
SP11	2	191–200	0	—	6	167–197	2	191–200	2	191–200
SP12	5	265–283	3	175–271	3	214–229	3	271–277	5	268–280
SP13	4	202–274	1	226	1	226	3	218–242	4	194–242
SP19	1	249	2	246–249	1	249	1	249	2	249–255
SP20	5	217–232	2	220–223	2	211–223	4	220–232	4	217–229
SP21	3	256–280	2	304–307	1	292	4	262–271	2	259–265
SP22	5	265–285	1	237	2	245–269	2	273–281	2	265–273
SP23	7	264–352	4	272–352	4	272–292	4	272–372	7	264–372
SP25	3	233–253	2	249–253	2	249–253	3	253–261	6	253–277
SP26	3	233–237	2	235–237	2	235–237	4	233–239	2	235–237
SP29	4	264–279	2	270–276	0	—	1	270	4	267–276
SP31	5	221–241	2	229–237	3	217–265	5	225–249	5	221–245
SP34	2	221–281	1	221	1	221	2	221–281	1	221
SP35	4	202–218	0	—	2	130–174	2	202–210	6	202–240

Note

— = unsuccessful amplification; *A* = number of alleles; *N* = number of individuals sampled.

a Locality and voucher information are provided in Appendix 1.

Cross‐amplification of the 21 primer pairs was conducted in five widely distributed congeneric species of *Saussurea* on the Korean peninsula. Three species (*S. gracilis*,* S. grandiflora*, and *S. tanakae*) were all successfully amplified and polymorphic for the 21 loci, while two species (*S. maximowiczii* and *S. pulchella*) were successfully amplified and polymorphic for 18 loci.

## CONCLUSIONS

The results of the present study represent the second set of SSR markers developed for the genus *Saussurea*. The 21 SSR markers developed in this study will be useful for studying genetic diversity, gene flow, and population structure in *S. polylepis*. In addition, the resulting population genetic studies will help to develop appropriate conservation strategies and management plans of the endangered *S. polylepis* on continental islands in Korea. Lastly, the successful cross‐amplification of these markers among commonly found *Saussurea* species in Korea will provide a novel population genetic tool in one of the most speciose groups in Korea.

## AUTHOR CONTRIBUTIONS

S.A.Y. and S.‐C.K. designed the project. S.A.Y. collected samples in the field, conducted the experiments, analyzed the data, and wrote the draft of the manuscript. S.‐C.K. supervised the study and revised the manuscript. All authors approved the final version of the manuscript.

## Data Availability

All sequence information was deposited in the National Center for Biotechnology Information (NCBI) Sequence Read Archive (BioProject ID PRJNA503488), and genomic DNA was deposited in the NCBI BioSample database (accession number SAMN10362162). Primer sequences were uploaded to GenBank and accession numbers are provided in Table 1.

## APPENDIX 1 Locality and voucher information of three *Saussurea polylepis* populations and five congeneric species used in this study.

**Table:**

Species	Collection locality	Geographic coordinates^a^	*N*	Voucher specimen^b^
*Saussurea polylepis* Nakai	Hongdo, Jeolla Province, Korea	—	13	SKKU044834
	Heuksando, Jeolla Province, Korea	—	20	SKKU044835
	Uido, Jeolla Province, Korea	—	15	SKKU044836
*Saussurea gracilis* Maxim.	Mt. Gaya, Gyeongsang Province, Korea	35.825868°N, 128.117077°E	1	SKKU044840
	Mt. Yongmoon, Gyeonggi Province, Korea	37.560846°N, 127.543898°E	1	SKKU044841
	Mt. Naejang, Jeolla Province, Korea	35.485817°N, 126.881233°E	1	SKKU044843
	Mt. Doksil, Jeolla Province, Korea	34.076889°N, 125.105500°E	1	SKKU044847
	JeJu island, Korea	33.358472°N, 126.503917°E	1	SKKU031085
*Saussurea grandifolia* Maxim.	Mt. Odae, Gangwon Province, Korea	37.802250°N, 128.547222°E	1	SKKU031089
	Mt. An, Gangwon Province, Korea	38.141981°N, 128.326502°E	1	SKKU031079
	Mt. Daeam, Gangwon Province, Korea	38.182150°N, 128.093567°E	1	SKKU031080
	Uleung island, Gyeongsang Province, Korea	37.513883°N, 130.883817°E	1	SKKU044837
	Russia	43.032722°N, 134.150306°E	1	SKKU044844
*Saussurea maximowiczii* Herder	Mt. Chilbo, Gyeonggi Province, Korea	37.258249°N, 126.933596°E	1	SKKU044838
	Mt. Bisle, Gyeongsang Province, Korea	35.714933°N, 128.525200°E	1	SKKU044846
	Mt. Baekun, Jeolla Province, Korea	35.067500°N, 127.646917°E	1	SKKU031088
	Mt. Mudeung, Jeolla Province, Korea	35.137000°N, 127.015000°E	1	SKKU031087
	JeJu island, Korea	33.358033°N, 126.462873°E	1	SKKU031078
*Saussurea pulchella* (Fisch.) Fisch. ex Colla	Mt. Hambaek, Gangwon Province, Korea	37.180667°N, 128.893850°E	1	SKKU044839
	Mt. Odae, Gangwon Province, Korea	37.792383°N, 128.563083°E	1	SKKU044853
	Mt. Goyang, Gangwon Province, Korea	37.414733°N, 128.752617°E	1	SKKU044852
	Bongwha, Gyeongsang Province, Korea	37.030500°N, 128.985611°E	1	SKKU044854
	Russia	43.031028°N, 131.556639°E	1	SKKU044845
*Saussurea tanakae* Franch. & Sav. ex Maxim.	Mt. Hwaak, Gyeonggi Province, Korea	37.990133°N, 127.500517°E	1	SKKU044842
	Mt. Hambaek, Gangwon Province, Korea	37.148183°N, 128.908917°E	1	SKKU044848
	Mt. Seorak, Gangwon Province, Korea	38.136000°N, 128.346967°E	1	SKKU044850
	Mt. Deokhang, Gangwon Province, Korea	37.325683°N, 129.003617°E	1	SKKU044851
	Mt. An, Gangwon Province, Korea	38.141981°N, 128.326502°E	1	SKKU044849

Note

*N* = number of samples.

a Geographic coordinates are not provided for *Saussurea polylepis* to due to the vulnerable status of the species.

b All voucher specimens are deposited in the Ha Eun Herbarium (SKK; Sungkyunkwan University, Korea).
